# Autism and the Oral Microbiome: A Systematic Review of Host-microbial Interactions and Diversity

**DOI:** 10.1016/j.identj.2025.100957

**Published:** 2025-09-09

**Authors:** Kausar Sadia Fakhruddin, Iman Kamal, Tatia Maglaperidze, Anand Marya, Lakshman Samaranayake, Thantrira Porntaveetus

**Affiliations:** a Faculty of Dentistry, The University of Western Australia, Nedlands, Western Australia, Australia; b Faculty of Dentistry, University of Saskatchewan, Saskatchewan, Canada; c Center of Excellence in Precision Medicine and Digital Health, Geriatric Dentistry and Special Patients Care International Program, Department of Physiology, Faculty of Dentistry, Chulalongkorn University, Bangkok, Thailand; d Georgian National University SEU, Faculty of Medicine, Georgia; e Faculty of Medicine, University of Georgia, Tbilisi, Georgia; f Faculty of Dentistry, University of Puthisastra, Phnom Penh, Cambodia; g Global Research Cell, Dr DY Patil Dental College and Hospital, Dr DY Patil Vidyapeeth, Pimpri, Pune, 411018, India; h Faculty of Dentistry, University of Hong Kong, 34, Hospital Road, Hong Kong; i Clinic of General – Special Care and Geriatric Dentistry, Center for Dental Medicine, University of Zurich, Zurich, Switzerland

**Keywords:** Dysbiosis, Healthcare, Well-being, Neurological disorder, Health disparity, Oral health

## Abstract

**Background:**

Emerging evidence suggests a link between the oral microbiome and autism spectrum disorder (ASD), a neurodevelopmental condition characterised by social and behavioural impairments. The vast microbial reservoirs in the gut complement those of the oral cavity, suggesting a potential oral-gut-brain axis that may influence ASD and perhaps other neurological diseases, such as Parkinson’s syndrome and Alzheimer’s disease. For the first time, this systematic review synthesises the current knowledge of oral microbiome composition, diversity, and functionality in ASD and its potential diagnostic and therapeutic implications.

**Methods:**

A comprehensive literature search was conducted using Medline (PubMed), Embase, Scopus, and Google Scholar for peer-reviewed case-control and cross-sectional studies published between January 2000 and January 2025. Study quality was assessed using the Newcastle–Ottawa scale.

**Results:**

Nine studies (n = 8533; 2536 ASD and 5937 controls) met the inclusion criteria. The overall findings on microbial diversity were inconsistent; some studies reported alterations in ASD, while others found no significant differences. Functional profiling revealed enrichment of pathways involved in dopamine and GABA degradation, as well as disruptions in lysine metabolism, suggesting possible links to neurotransmitter imbalances in ASD. Although external factors such as selective eating, oral hygiene, and cognitive function were proposed to influence microbial profiles, statistical evidence supporting these associations was lacking. Moreover, no consistent link was found between oral microbiota features and core ASD symptoms like repetitive behaviours or communication deficits.

**Conclusion:**

This review highlights subtle yet potentially significant alterations in the oral microbiome of individuals with ASD, particularly in metabolic pathways that affect neurotransmitters. While direct associations with clinical symptoms remain unsubstantiated, the findings emphasise the importance of future multi-omics and longitudinal studies to clarify the oral microbiome’s role in ASD pathophysiology and to explore its potential in personalised therapeutic strategies.

## Introduction

Autism spectrum disorder (ASD) impacts brain development and influences an individual’s communicative abilities and interactive social behaviours. The variability inherent in ASD manifests as a broad spectrum of clinical presentations, with individuals requiring differing levels of support as defined by the DSM-5, ranging from Level 1 (requiring support) to Level 3 (requiring very substantial support). This is based on the severity of social communication deficits and restrictive, repetitive behaviors.^1^^,^^2^ The ethology of autistic traits remains elusive despite extensive research and various theories.

Individuals with autism often exhibit a tendency towards a limited range of interests and repetitive behaviours. Food selectivity, while also present in typically developing children at younger ages, tends to be more pronounced and restrictive in those with ASD. This selectivity can vary in frequency and severity among affected individuals.^3^ The underlying mechanisms of this selectivity are not fully understood, but several links to various factors have been proposed. Undiagnosed subclinical autistic characteristics may secondarily influence family-based eating habits, such as restrictive dietary choices and monotonous behaviours among parents, leading to the adoption of food preferences not unlike those with ASD.^3^^,^^4^

ASD is associated with several oropharyngeal abnormalities, including buccal sensory sensitivity, taste and texture aversions, and salivary transcriptome alterations. Empirical evidence indicates that food-specific oral sensory peculiarities in autistic individuals contribute to atypical eating behaviours, such as dysgeusia and heightened sensitivity to the taste, texture, smell, and appearance of food, which often results in restricted and less diverse diets.^5^ Chistol and colleagues,^6^ found that children on the autism spectrum with atypical oral sensory sensitivity were more likely to reject a wide range of foods and had lower intake of fibre-rich vegetables and fruits compared to those with typical oral sensations. These dietary limitations are likely to disrupt the balance of the oral and gut microbiota.^7^^,^^8^ Additionally, the autonomic nervous system plays a critical role in regulating emotional responses and eating behaviors.^9^ In individuals with ASD, unpleasant sensory experiences, impaired muscular coordination, or untreated dental caries can further hinder chewing and oral function, exacerbating dietary restrictions and potentially impacting the composition of the oral and gut microbiome^10^^,^^11^ possibly impacting the oral and gut microbiome.^12^

Furthermore, the phenomenon of alexithymia, characterised by difficulties in recognising and expressing emotions and bodily sensations, along with an imagination deficit, may intersect with food preference and feeding issues in children with ASD.^13^

Oral hygiene is another critical factor in maintaining the eubiotic ecosystem of the oral microbiome and, by extension, influencing gut microbiota through the oral-gut axis. Poor oral hygiene can lead to oral dysbiosis, contributing to common oral diseases such as caries, gingivitis, and periodontitis.^14^ Consequently, oral pathogens and inflammatory molecules, such as interleukins, can translocate to the gut via the salivary route or indirectly through the systemic route, potentially leading to a dysbiotic gut microbiome.^15^^,^^16^ This risk is heightened in individuals with ASD, who often face challenges in maintaining consistent oral hygiene and managing the aforementioned consequences.^17^ Thus, poor oral hygiene in ASD can exacerbate microbial imbalances, potentially leading to worsening of oral and gut health.

Emerging research has highlighted the potential role of the microbiome, notably the gut microbiome, in the pathophysiology of ASD.^18^ The oral cavity, an extension of the digestive tract, harbours one of the most diverse microbial communities in the human body, second only to the gut. Its abundant microbiome includes more than 700 identified bacterial species.^19^ Given that the oral cavity represents the entry point to the gastrointestinal tract, the concept of a microbial oral-brain axis is an extension of the well-established gut-brain axis.^19^ However, the oral microbiome, has received comparatively less attention than the gut microbiome despite its potential impact on systemic health and neurological function. Recent studies have suggested that dysbiosis in the oral microbiota may be linked to various neurological disorders, including Alzheimer’s disease, epileptic seizures, multiple sclerosis, migraines, and Parkinson’s disease.^20^

Despite the growing body of data on the oral microbiome of individuals with ASD, critical gaps remain in our understanding of their oral microbiome. While specific studies have documented differences in oral microbial diversity in ASD,^21^^,^^22^ there has been no comprehensive review synthesising the collective evidence to clarify whether dietary habits, oral hygiene practices, or other environmental determinants significantly impact the condition. This systematic review, therefore, aims for the first time to characterise the oral microbiome in individuals with ASD, identify microbial diversity as well as functional and metabolic profiles, and assess the factors that modulate these microbial shifts. By addressing these gaps, we aim to lay the groundwork for future research into microbiome-targeted interventions and their potential impact on ASD symptomatology.

## Methodology

A systematic scoping review was conducted to identify English-language articles documenting children and young adults diagnosed with ASD. The review primarily included case-control and cross-sectional studies published in peer-reviewed journals. To retrieve relevant studies published between January 2000 and January 2025, a comprehensive search was performed using Medline (via PubMed), Google Scholar, Embase, and Scopus databases.

### Search terms

#### Medline (via PubMed)

("Autism Spectrum Disorder"[MeSH] OR "Autism"[MeSH] OR "ASD"[TIAB] OR "Neurodevelopmental Disorder"[TIAB] OR "Autistic children"[TIAB])
AND
("Oral microbiome"[MeSH] OR "Oral microbiota"[TIAB] OR "Salivary microbiome"[TIAB] OR "Salivary microbial diversity"[TIAB] OR "Dental plaque microbiome"[TIAB] OR "Tongue microbiome"[TIAB])
AND
("16S rRNA sequencing"[MeSH] OR "Metagenomics"[MeSH] OR "Shotgun sequencing"[TIAB] OR "Microbial profiling"[TIAB] OR "High-throughput sequencing"[TIAB])
AND
("Microbial diversity"[MeSH] OR "Bacterial composition"[TIAB] OR "Dysbiosis"[TIAB] OR "Microbial metabolism"[TIAB])

#### Google Scholar

"Autism Spectrum Disorder" OR "ASD" OR "Autistic children" OR "Neurodevelopmental disorder"
AND
"Oral microbiome" OR "Oral microbiota" OR "Salivary microbiome" OR "Dental plaque microbiome" OR "Tongue microbiome"
AND
"16S rRNA sequencing" OR "Metagenomic sequencing" OR "Shotgun sequencing" OR "Microbial composition" OR "Microbial diversity"
AND
"Microbial metabolism" OR "Dysbiosis" OR "Functional pathways"

#### Embase

('autism spectrum disorder'/exp OR 'autism'/exp OR 'ASD':ti,ab OR 'neurodevelopmental disorder':ti,ab OR 'autistic children':ti,ab)
AND
('oral microbiome'/exp OR 'oral microbiota'/exp OR 'salivary microbiome':ti,ab OR 'dental plaque microbiome':ti,ab OR 'tongue microbiome':ti,ab)
AND
('16S rRNA sequencing'/exp OR 'metagenomic sequencing':ti,ab OR 'shotgun sequencing':ti,ab OR 'microbial diversity':ti,ab OR 'microbial composition':ti,ab)
AND
('microbial metabolism'/exp OR 'dysbiosis'/exp OR 'functional pathways':ti,ab)

#### Scopus

(TITLE-ABS-KEY("Autism Spectrum Disorder") OR TITLE-ABS-KEY("ASD") OR TITLE-ABS-KEY("Autistic children") OR TITLE-ABS-KEY("Neurodevelopmental disorder"))
AND
(TITLE-ABS-KEY("Oral microbiome") OR TITLE-ABS-KEY("Oral microbiota") OR TITLE-ABS-KEY("Salivary microbiome") OR TITLE-ABS-KEY("Dental plaque microbiome") OR TITLE-ABS-KEY("Tongue microbiome"))
AND
(TITLE-ABS-KEY("16S rRNA sequencing") OR TITLE-ABS-KEY("Metagenomic sequencing") OR TITLE-ABS-KEY("Shotgun sequencing") OR TITLE-ABS-KEY("Microbial profiling") OR TITLE-ABS-KEY("Microbial diversity"))
AND
(TITLE-ABS-KEY("Microbial metabolism") OR TITLE-ABS-KEY("Dysbiosis") OR TITLE-ABS-KEY("Functional pathways"))

#### Focused question

What are the key characteristics of the oral microbiome in individuals with ASD, and if external factors influence its composition and functional pathways?

#### Primary outcome

To identify and summarise the key findings related to microbial diversity, species abundance, and functional pathways in the oral microbiomes of individuals with ASD.

#### Secondary outcome

To summarise external factors, such as diet, oral hygiene, comorbidities, and cognition, that may influence the oral microbiome in ASD, and to highlight emerging trends and hypotheses.

### Study selection

#### Inclusion criteria

##### Study population

Studies involving individuals diagnosed with ASD based on recognised diagnostic criteria (Diagnostic and Statistical Manual of Mental Disorders, 5th edition (DSM-5), International Classification of Diseases, 10th revision (ICD-10), or professional clinical diagnosis).

Studies include a control group of neurotypical individuals or individuals with other developmental disorders for comparison.

##### Study design

Case-control studies, cross-sectional studies, cohort studies.

##### Microbiome analysis

Studies that use oral or oral and gut microbiological assessment.

##### Sample type

Studies analysing oral microbiome from saliva, tongue scraping, dental plaque, or other oral samples.

##### Language

Peer-reviewed studies published in English.

#### Exclusion criteria

1. Non-human and non-English studies.
2. Studies focusing only on the gut microbiome.
3. Lack of ASD diagnosis or control group.
4. Studies that do not provide a precise ASD diagnosis based on DSM-5/ICD-10 criteria.
5. Studies that do not include a control group.
6. Intervention-based studies: Clinical trials assess the effects of probiotics, prebiotics, antibiotics, or other therapeutic interventions on the oral microbiome rather than characterising microbial differences.
7. Narrative reviews, systematic reviews, meta-analyses, commentaries, editorials, letters to the editor, and conference proceedings without original data.
8. Studies that do not report sufficient details on the ASD group, control group, sample size, or microbiome composition.
9. Studies with methodological flaws or high risk of bias (eg, lack of microbial sequencing techniques, non-standardised diagnostic criteria).

### Electronic data search, data extraction, and analysis

**Fig. 1:**
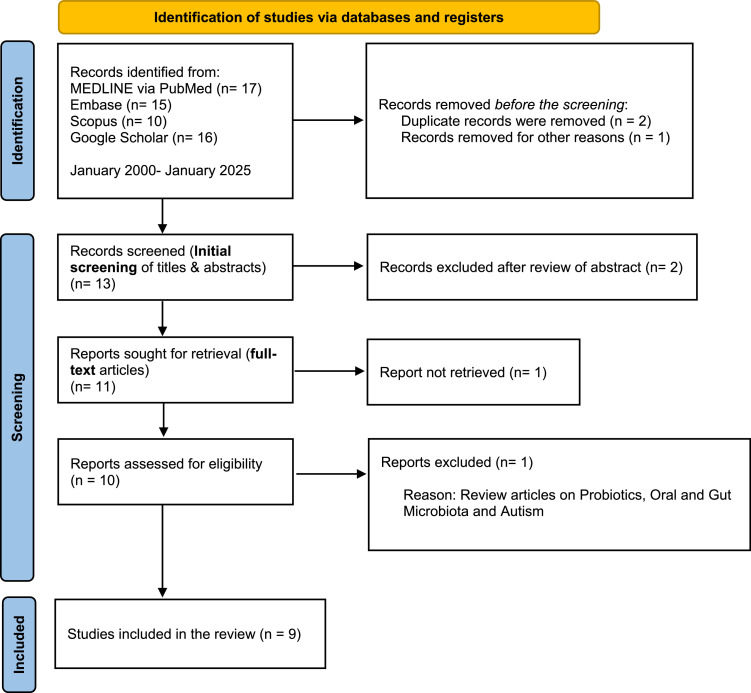
PRISMA flow chart of the literature search and study selection.

This systematic review followed the Preferred Reporting Items for Systematic Reviews and Meta-Analyses for Scoping Reviews (PRISMA-ScR) guidelines,^23^ to ensure a rigorous and standardised methodological approach. Due to the heterogeneity in study design, sample sizes, and microbial assessment methods among the included studies, a meta-analysis is not feasible. The variability in methodologies limits direct comparisons and prevents the derivation of a single, quantifiable effect size. The present review adopts a scoping systematic approach to comprehensively map the existing literature rather than synthesising a single statistical outcome. Doing so ensures that the complexity and diversity of findings are accurately represented. Figure 1 provides a summary of the search strategy and study selection process.

The electronic data search and analysis were conducted in 3 sequential steps. Initially, 2 independent reviewers (KSF and IK) screened the titles and abstracts of all identified studies based on predefined inclusion criteria. A third reviewer (TP) independently reviewed the discrepant items. A consensus was achieved through discussions among all reviewers. If sufficient information for eligibility assessment was unavailable in the abstract, a full-text review was performed. The selected studies were critically evaluated against the eligibility criteria during this stage, and relevant data were systematically extracted and documented. To enhance the comprehensiveness of the review, we manually searched the reference lists of included studies to identify any additional relevant publications.

Data extraction and assessment were performed in the final stage, documenting key study characteristics, including study design, participant demographics, and sample type (saliva, dental plaque, and tongue microbiome) (Table 1). The molecular techniques used to analyse oral microbiota, microbial diversity metrics, and functional pathway analyses are presented in Table 2. Information regarding potential external factors and broader environmental influences was also systematically analysed. All identified studies meeting the inclusion criteria were organised and managed using EndNote version 11 (Clarivate Analytics) to facilitate structured reference management and data retrieval.

**Table 1:** Characteristics of the included studies.

Study Country Study design	N (ASD) Age (ASD, mean ± SD or range) Female Male	N (Control) Age (ASD, mean ± SD or range) Female Male	Diagnosis of autism	Comorbidity
Tang et al^30^ Hong Kong Cross-sectional	N = 25 3–6 y (Mean: 4.79 ± 0.83) Female = 6 Male = 19	N = 30 Age: 3–6 y (Mean: 4.90 ± 0.85) Female = 6 Male = 24	DSM-5 criteria	No significant reported comorbidities The study controlled for potential confounders such as dietary frequency, milk intake, and birth weight. Caries prevalence was reported as lower in ASD children than in TD children.
Evenepoel et al^31^ Belgium Case-control	N = 80 8–12 y (Mean: 10.5 ± 1.3) Female = 16 Male = 64	N = 40 Age: 8–12 y (Mean: 10.3 ± 1.3) Female = 8 Male = 32	DSM-5 criteria Additional confirmation using Autism Diagnostic Observation Schedule-2 (ADOS-2)	Participants had an IQ > 70 (measured using the Wechsler Intelligence Scale for Children, Fifth Edition) Higher levels of anxiety and repetitive behaviours compared to non-autistic children No significant neurological disorders (stroke, epilepsy, concussion, etc.) Excluded children with significant physical disorders (liver, renal, cardiac pathology) Excluded children who had used antibiotics in the last 3 mo
Manghi et al^22^ United States Cross-sectional	N = 2154 9 y (range 8.6–9.1) Female = 411 Male = 1743	N = 1646 (NT siblings) 8 y (range 7.8–8.2) Female = 858 Male = 788	Professional ASD diagnosis through the SPARK consortium	Cognitive impairment (IQ < 70) Dietary habits (restrictive eating) Developmental coordination disorder
Oda et al^25^ Turkey Case-control	N = 9 36–60 mo (Mean: 44 ± 6.34 mo) Female = 0 Male = 9	N = 9 Age-matched (Mean: 46.89 ± 7.99 mo) Female = 0 Male = 9	DSM-5 criteria	None were reported. Exclusions included gastrointestinal disorders, ADHD, and chronic systemic diseases.
Abdulhaq et al^29^ Saudi Arabia Case-control	N = 25 9.24 ± 1.96 y Female = 9 Male = 16	N = 38 10.03 ± 1.48 y Female = 20 Male = 18	DSM-5 criteria	Clinical examination: dmft/DMFT; BI Exclusion includes gingival bleeding on probing in more than 10 of the sites; a history of using antimicrobials and/or steroids within the last 3 mo
Forsyth et al^28^ United States Case-control (pilot study)	N = 11 10.68 y (range 8.67–13 y) 90.9% of the ASD group were boys, likely (1 girl and 10 boys)	N = 10 Aged 6–15 y NM	DSM-5 criteria	*Gastrointestinal disturbances*: Gastroesophageal reflux, constipation, chronic diarrhoea, abdominal pain *Intellectual impairments* *Language deficits* *Motor deficits* *Sensory abnormalities and pain sensitivity*
Kong et al^26^ United States First-degree relative matched design	N = 20 7-25 y Female = 5 Male = 15	N = 19 11–50 y Female = 11 Male = 8	DSM-5 criteria	*Allergies* *Gastrointestinal disturbances and dietary habits*
Hicks et al^27^ United States Case-control	N = 180 Mean age 53 ± 16 mo Female = 26 Male = 154	N = 106 Mean age of 43 ± 16 mo Female = 42 Male = 64 *Nonautistic developmental delay:* N = 60 Mean age of 50 ± 13 mo Female = 17 Male = 43	DSM-5 criteria	*Gastrointestinal disturbances:* Constipation, chronic diarrhoea, abdominal pain, gastroesophageal reflux *Attention deficit hyperactivity disorder*
Qiao et al^21^ China Cross-sectional	N = 32 7–14 y (Mean: 10.02 ± 1.43 y) Female = 5 Male = 27	N = 27 Age-matched (Mean: 10.19 ± 0.59 y) Female = 6 Male = 21	DSM-5 and ICD-10 criteria	None were reported. Exclusions included prior antibiotic/antifungal use and gastrointestinal disorders.

NM, not mentioned.

**Table 2:** Microbial composition and key differences in the oral microbiome of individuals with autism spectrum disorder and neurotypical controls.

Study	Samples Sequencing platform	DNA extraction method Primers	Alpha diversity Beta diversity	Oral microbial composition (key microbial differences)
Tang et al^30^ Hong Kong	Dental plaque Illumina NovaSeq PE250	QIAamp DNA Mini Kit 16S V3–V4	Chao1 significantly lower in ASD (*P* = .035) Significant (NMDS UniFrac, *P* = .016)	Lower bacterial diversity in ASD children compared to TD controls **Six discriminatory species enriched in ASD:** *Microbacterium flavescens* *Leptotrichia sp. HMT-212* *Prevotella jejuni* *Capnocytophaga leadbetteri* *Leptotrichia sp. HMT-392* *Porphyromonas sp. HMT-278* **Five discriminatory species enriched in TD controls:** *Fusobacterium nucleatum* subsp. *polymorphum* *Schaalia sp. HMT-180* *Leptotrichia sp. HMT-498* *Actinomyces gerencseriae* *Campylobacter concisus*
Evenepoel et al^31^ Belgium	Tongue swabs Illumina MiSeq 2 × 300 bp	RNeasy PowerMicrobiome Kit 16S V3–V4 (341F/785R)	No significant difference (all *P* > .05) No significant difference (ANOSIM *P* = .866)	No significant difference in alpha and beta-diversity **Five bacterial genera significantly enriched in ASD:** *Solobacterium* *Stomatobaculum* *Ruminococcaceae UCG.014* *Tannerella* *Campylobacter* No significantly enriched bacterial genera were found in TD children
Manghi et al^22^	Saliva Illumina NovaSeq 6000	Chemomagic MSM1/360 (PCR-free) N/A (Shotgun metagenomics)	No significant difference (*P* > .05) Significant (PERMANOVA *P* = .001; R² = 2%)	**Distinct microbial signatures in ASD vs NTs:** 108 bacterial species significantly differed between ASD children and NTs. 52 species were more abundant in ASD, 56 species were more abundant in NTs. **Microbes enriched in ASD:** The aerotolerant bacterium *Rothia dentocariosa* was significantly prevalent in ASD, followed by *Actinomyces hongkongensis, A. johnsonii, Cutibacterium acnes, C. durum, Eikenella sp*. NML 130454, *Streptococcus gordonii* **Microbes enriched in NTs:** The genus *Prevotella* was particularly associated with NTs, comprising 12 species None was associated with ASD **Host-microbiota functional metabolic implications** Increased GABA and dopamine degradation enzyme potential
Oda et al^25^	Saliva Oxford Nanopore MinION	ZymoBIOMICS DNA Miniprep Kit 16S V1–V9 (27F/1453R)	Higher in ASD but not significant No significant difference (PCoA)	**Microbial composition in ASD vs neurotypical controls** There are no significant differences in overall alpha and beta diversity Firmicutes are the most abundant phylum in both groups: ASD (73.68%) vs Controls (80.30%) *Streptococcus* was the dominant genus in both groups: ASD (57.2%) vs Controls (65.71%) Proteobacteria (11.57%) and Bacteroidetes (5.45%) were higher in ASD vs Controls (7.65% and 2.52%, respectively)
Abdulhaq et al^29^	Tongue scraping Illumina MiSeq 2 × 300 bp	PureLink Genomic DNA Kit 16S V1–V3 (27FYM/519R)	No significant difference No significant difference (PCA)	There are no significant differences in overall alpha and beta diversity **ASD vs TD** *Actinomyces odontolyticus* and *A.* *Lingnae.* Depletion of *Campylobacter concisus* and *Streptococcus vestibularis* in the ASD group versus TD
Forsyth et al^28^	Saliva Illumina MiSeq	ORAcollectDNA + NucleoSpin RapidLyse 16S V3–V4 (338F/806R)	Not statistically tested beyond rarefaction PCoA indicated significant differences	**Higher in ASD vs TD** *Rothia* species (12.2-fold increase) compared to typically developing children In boys with ASD specifically, *Rothia* exhibited a 17.8-fold change. **Higher in TD vs ASD children** *Megasphaera* (39.2-fold decrease in ASD) *Moraxella* (31.9-fold decrease in ASD) *Neisseria* (18.8-fold decrease in ASD) *Gemella* (14.0-fold decrease in ASD) In boys with ASD *Moraxella* and *Neisseria* were paradoxically increased compared to ASD girls and TD children (42.36-fold and 28.62-fold increases, respectively)
Kong et al^26^	Saliva Illumina MiSeq 2 × 250 bp	HR-Easy Fecal DNA Kit 16S V3–V4 (341F/805R)	No significant difference No significant difference (PERMANOVA *P* > .05)	No significant differences in overall bacterial diversity (alpha diversity) and microbial composition differences (beta diversity): between the ASD and control groups. Lower relative abundance of TM7 bacteria and altered levels of Bacilli species in ASD patients **Gut microbiota differences** Increased Firmicutes/Bacteroidetes ratio Higher abundance of Proteobacteria Increased *Butyricimonas* Differential abundance of genera *Paraprevotella, Granulicatella, Butyricimonas, Peptoniphilus*, and *Eubacterium*
Hicks et al^27^	Saliva Illumina NextSeq 500	RNA extraction (Trizol & RNeasy) N/A (Shotgun metatranscriptomics)	No significant difference (Shannon *P* = .60) Significant (Bray-Curtis, *P* = .04)	A total of 28 taxa were identified that distinguished ASD patients with and without GI disturbance. **ASD vs TD** Increased in ASD: *Cyanobacteria* (FC = 2.38) *Limnohabitans* sp. 63ED37-2 (FC = 1.05) Planctomycetales (FC = 1.21) Decreased in ASD: *Ramlibacter tataouinensis* (FDR = 0.001) *Mucilaginibacter* sp. (FDR = 0.001) *Bacteroides vulgatus* (FDR = 0.05) *Gemmata* sp. SH-PL17 (FDR = 0.05) Dietary restrictions, food/medicine allergies, probiotic use, and vaccination status showed no correlations with oral taxonomic concentrations. **Host-microbiota functional metabolic implications** Increased oxidative phosphorylation and energy metabolism genes associated with *Cyanobacteria* Oxidative phosphorylation was 1.6 times more active in ASD children. Methane metabolism was 1.2 times more active in ASD children.
Qiao et al^21^	Saliva and dental plaque Illumina MiSeq 2 × 250 bp	OMEGA-soil DNA Kit 16S V3–V4 (338F/806R)	Dental: significantly lower in ASD (*P* < .05); Saliva: NS Significant difference in both habitats (PERMANOVA *P* < .01)	**Lower microbial diversity in ASD vs neurotypical controls:** ASD children exhibited significantly lower bacterial diversity, particularly in dental plaque samples. Distinct bacterial communities between ASD and controls **Key microbial differences between saliva and plaque samples in ASD:** Saliva: A higher abundance of *Haemophilus* Dental Plaque: Increased levels of *Streptococcus* **Commensal microbes decreased in ASD:** Saliva: *Actinomyces, Porphyromonas,* *Fusobacterium* Dental Plaque: *Prevotella, Selenomonas, Fusobacterium*

BI, bleeding on probing; FC, fold change; FDR, false discovery rate; N/A, not applicable; NS, not significant.

### Quality and the overall risk of bias assessment of the included reports

The methodological quality of the included case-control and cross-sectional studies was assessed by 2 reviewers (IK and TM) using the Newcastle–Ottawa scale,^24^ a validated tool for assessing the risk of bias in observational studies. The NOS evaluates studies based on 3 main domains: selection (representativeness of the ASD and control groups, method of diagnosis, and sample selection); comparability (matching or adjustment for confounding factors such as age, gender, diet, and comorbidities); outcome/exposure assessment (microbiome sampling method, sequencing techniques, and reliability of microbial diversity assessments).

Each study was assigned a quality score ranging from 0 to 9 points, with higher scores indicating a lower risk of bias. Studies scoring 6 or more points were considered moderate to high methodological quality, while those scoring 5 or below were categorised as having a higher risk of bias. Table 3 summarises the risk of bias assessment for the included studies.

**Table 3:** Risk of bias assessment of the included studies.

Study Total score	Selection				Comparability	Outcome	
	Representation of the sample	Sample size justification	Non-respondents’ description and analysis	Ascertainment of the exposure (validity and reliability of measurement)	Control for imp. confounding factors	Assessment of the outcome (validity and reliability of measurement)	Statistical test appropriate-ness and reporting
Tang et al^30^	**	*			*	*	*
Total score	6						
Evenepoel et al^31^	*	*		*	*	*	*
Total score	6						
Manghi et al^22^	**	**		*	*	*	*
Total score	8						
Oda et al^25^	**			*	*	*	*
Total score	6						
Abdulhaq et al^29^	**			*		*	*
Total score	5						
Forsyth et al^28^	**	*			*	*	*
Total score	6						
Kong et al^26^	**	*		*	*	*	*
Total score	7						
Hicks et al^27^	**		*	*	*	*	*
Total score	7						
Qiao et al^21^	**			*	*	*	*
Total score	6						

Within Newcastle-Ottawa Scale (NOS), each criterion in a section is awarded one asterisk when fulfilled. Some sections allow for more than one asterisk as they include multiple sub-criteria that can be satisfied.

The total score ranges from **0 to 9 stars**, with higher scores indicating a lower risk of bias.

## Results

### Study characteristics

The systematic review included 9 studies,^21^^,^^22^^,^^25^, ^26^, ^27^, ^28^, ^29^, ^30^, ^31^ encompassing varying study designs, including case-control studies, cross-sectional studies, and a first-degree relative matched design. The studies were conducted across diverse global regions, with combined studies consisting of (n = 8533) individuals, including ASD cases (n = 2536), neurotypical controls (n = 5937), and children with developmental delays (n = 60). The included studies examined children, adolescents, and young adults with ASD, with age ranges varying from 36 months to 25 years.

Among the ASD groups, there were more male participants (n = 1992) than female participants (n = 664) with ASD. The neurotypical control group (n = 1945) included 1175 males and 770 females.

All included studies employed the DSM-5 criteria,^32^ with one study,^21^ additionally incorporating ICD-10 in conjunction with DSM-5. Moreover, Manghi et al^22^ utilised a professional ASD diagnosis facilitated through the SPARK (Simons Foundation Powering Autism Research for Knowledge) consortium. Diagnostic criteria were assessed through standardised clinical assessments, including structured interviews and validated diagnostic instruments.

In many studies, a high prevalence of multisystem comorbidities was reported among individuals with ASD. Commonly documented comorbid conditions included gastrointestinal disturbances (eg, reflux, constipation, diarrhoea, abdominal pain), sensory processing abnormalities, intellectual and motor impairments, developmental coordination disorder, and attention-deficit/hyperactivity disorder (ADHD). The characteristics of the included studies are presented in Table 1.

### The oral microbiome in ASD: *Species-level composition and diversity*

The reviewed studies have attempted to explore the potential link between the oral microbiome and ASD, evaluating whether alterations in the oral microbiome may be associated with ASD. This is a relatively unexplored area compared to the search into the role of the gut microbiome in neurological conditions. Most of the reviewed studies,^25^^,^^26^^,^^29^, ^30^, ^31^ reported no significant differences in overall microbial diversity between ASD and neurotypical groups. However, our findings generally indicate that the oral microbiome in children with ASD exhibits subtle, species-level differences, with specific microbial taxa being either enriched or depleted, rather than a large-scale shift in overall core microbial diversity.

Certain bacterial taxa, particularly *Rothia* spp., were consistently enriched in salivary samples from the ASD group at the genus and species levels.^21^^,^^22^^,^^28^ Manghi et al^22^ noted a higher abundance of aerobic species characterised the ASD oral microbiome, whereas anaerobic species were more commonly associated with neurotypical individuals. Specifically, only 6 out of 56 aerotolerant species were linked to neurotypicals, while ASD children showed enrichment in aerotolerant species (25 out of 52). This shift is consistent with the increased abundance of species such as *Rothia dentocariosa, Streptococcus gordonii, Corynebacterium. durum,* and *Cutibacterium acnes* in ASD.

In a pilot study conducted by Forsyth et al,^28^ salivary samples from children with ASD revealed that *Rothia* (unclassified) was the predominant species in their salivary microbiota compared to neurotypic counterparts. Moreover, Qiao et al,^21^ also reported *Rothia* species in high amounts in dental plaque samples from ASD children but was much less common in their saliva. They reported that *Rothia mucilaginosa* was notably lower, while *Rothia aeria* was significantly higher in dental and saliva samples from ASD children.

Two of the reviewed studies^29^^,^^31^ provide complementary insights into the oral microbiome through analyses of the lingual scrapings of individuals with ASD and neurotypical controls. Both studies identified *Campylobacter concisus* as a key species of interest, though its abundance varied between the studies. Abdulhaq et al^29^ found the latter species depleted in ASD, while Evenepoel et al^31^ reported its enrichment in ASD. While these findings highlight the complexity of the oral microbiome analyses in ASD, they suggest that species-level differences, rather than the core microbial diversity, may better reflect a dysbiotic oral microbiome in ASD.

Two additional key studies^21^^,^^30^ have investigated the oral microbiome in children with ASD using differing microbial samples, dental plaque, either with or without saliva, and have consistently observed reduced microbial diversity, especially in plaque samples. Qiao et al^21^ also observed distinct microbial communities in dental plaque samples from ASD children, with increased levels of *Streptococcus* and decreased levels of commensal microbes such as *Prevotella, Selenomonas*, and *Fusobacterium*. While Tang et al did not mention *Streptococcus* as a key difference. Both studies highlight a reduction in beneficial commensal bacteria in ASD children, possibly contributing to dysbiosis of the oral microbiome.

Kong et al^26^ conducted a seminal study comparing the oral and gut microbiomes using RNA sequencing in individuals with ASD and neurotypical individuals. They were unable to find any significant differences in either alpha or beta diversity of the microbiome between the salivary and gut microbial profiles in either the ASD or control groups. However, they noted an unspecified oral *Bacilli* genus, the relative abundance of which significantly differed between the ASD and control groups. Additionally, they observed the co-occurrence of specific clades of gut and oral microbial taxa despite the anatomical separation of the ecosystems. For instance, a strong correlation was found between gut *Firmicutes* and saliva *Chloroflexi* in the ASD population, suggesting potential interactions between these distant microbial communities.

Hicks et al’s^27^ noted in another metatranscriptomic analysis of the salivary microbiome identified a striking abundance of *Cyanobacteria* in ASD patients compared to neurotypical controls, with a 2.38-fold increased prevalence of the genus. This observation is particularly intriguing given the traditionally non-oral nature of *Cyanobacteria*, which are commonly found in aquatic environments. These variances may reflect differences in the samples analysed, such as saliva versus plaque, or the sampling technique *itse*lf, or population heterogeneity. Table 2 presents the microbial composition and highlights key differences in the oral microbiome between individuals with ASD and neurotypical controls.

### Correlation between oral microbiota and ASD phenotypes

The reviewed studies revealed no robust correlation between the oral microbiota and core phenotypic features of ASD, such as repetitive behaviours and communication impairments.

### Influence of clinical comorbidities and environmental factors on the oral microbiome in ASD

Although gastrointestinal disturbances and sleep difficulties are commonly reported comorbidities in individuals with ASD, the reviewed studies provided limited data directly correlating these symptoms with oral microbiome profiles. Similarly, while several investigations acknowledged the potential impact of external factors, such as selective dietary habits and inconsistent oral hygiene practices on microbial composition, robust statistical analyses linking these influences on microbial dysbiosis were largely absent. Notably, Manghi et al^22^ reported that children with ASD and IQ scores below 70 exhibited significantly lower strain-level oral microbiome similarity with their parents compared to their neurotypical siblings. This observation suggests that cognitive function, independent of hygiene or dietary factors, may have a nuanced effect on the oral microbial composition.

### The oral-brain axis in ASD: Functional metabolic pathways in ASD oral microbiome

Hick et al^27^ observed significant differences in microbial functional pathways related to energy metabolism and lysine degradation, indicating distinct metabolic profiles in children with ASD compared to typically developing and developmentally delayed controls. Similarly, Manghi et al^22^ reported that the oral microbiota in the ASD group exhibited functional potential for degrading neurotransmitters such as dopamine and gamma-aminobutyric acid (GABA), along with elevated read counts for the enzymatic pathway involved in the conversion of serotonin to 5-hydroxytryptophan. These findings suggest an association between altered microbial functional pathways and potential cognitive processes in ASD.

## Discussion

There is an accumulating database to indicate that the properties of the resident human microbiome, including the oral microbiome, could be reflective of the neurodevelopmental attributes of their hosts.^33^ This review offers the first comprehensive synthesis of studies investigating the oral microbiome in those with ASD, aiming to determine whether distinct microbiota profiles are associated with the condition.

Microbial colonisation begins at birth, with the mode of delivery of the infant (vaginal vs cesarean) playing a role in modulating the development of the early microbiome. However, studies on the association between delivery mode and ASD have yielded mixed results, suggesting that early microbial dysbiosis is likely influenced by multiple factors rather than a single determinant.^34^, ^35^, ^36^ Of particular interest is the finding by Manghi et al^22^ that children with ASD and lower IQ scores exhibit significantly lower microbiome strain-sharing with their parents, indicating disruptions in microbial transmission and/or colonisation. This suggests that cognitive function may impact microbial composition, as children with lower IQs (≤70) often experience more severe feeding difficulties, including strong food aversions, heightened sensory sensitivities, and rigid dietary preferences.

Challenges that lead to restricted dietary diversity are likely to limit the nutrients supporting the growth of microbiota.^37^ Additionally, difficulties in adaptive behaviours, such as maintaining consistent oral hygiene or engaging in food-sharing activities, may further impact microbial diversity and contribute to dysbiosis. However, whether this pattern differs in children with higher IQs (>70) remains unclear, as Manghi’s study,^22^ did not explicitly confirm the reverse trend. While it is generally assumed that children with higher IQs may exhibit greater flexibility in food selection and dietary patterns, potentially supporting a more diverse microbiome, this assumption requires further validation.^22^^,^^38^ Future research should investigate how cognitive function modulates dietary behaviours and, in consequence, microbiota composition, as well as the efficacy of interventions to improve oral and systemic health outcomes in children with ASD.

The enrichment of Cyanobacteria in the oral microbiome of children with ASD is particularly intriguing, given its non-oral nature and potential neurotoxic effects. Cyanobacteria are saprophytic organisms usually found in stagnant water, such as lakes, where they reproduce exponentially to form blooms.^39^ Cyanobacteria are known to produce cyanotoxins, such as β-N-methylamino-L-alanine (BMAA), which is implicated in neurodegenerative diseases through mechanisms including neuroinflammation, oxidative stress, and excitotoxicity.^40^ These toxins may cross the blood-brain barrier and disrupt neuronal function, potentially contributing to the neurodevelopmental dysfunction observed in ASD. For instance, BMAA can mimic glutamate, leading to excessive activation of glutamate receptors and subsequent excitotoxicity,^41^ a process linked to synaptic dysfunction and possible neural hyperactivity in ASD. There are also reports of cyanotoxins inducing mitochondrial dysfunction and activating the immune system,^42^ further exacerbating neuroinflammation and oxidative stress.

Moreover, the presence of cyanobacteria as noteworthy constituents of the oral microbiota may also reflect broader systemic dysregulation, as they have been associated with affections, such as gastrointestinal disturbances, hay fever, and pruritus^43^ commonly reported in ASD. Taken together, these observations suggest that oral cyanobacteria could play a role in ASD pathophysiology through direct neurotoxic effects and systemic inflammation. Further investigations are therefore warranted to examine their mode of action and potential for therapeutic targets.

Mouth breathing is an often-overlooked factor that significantly influences the composition of oral microbiota. Independent studies have highlighted a higher incidence of upper respiratory affections in children with ASD, which likely contributes to mouth breathing.^44^ Several reviewed studies,^21^^,^^22^^,^^28^ reported an increased prevalence of aerotolerant microbes in the oral microbiome of children with ASD, suggesting a potential link between microbial composition and altered respiratory profiles.

Mouth breathing alters the oral microenvironment by reducing salivary flow, increasing oxygen exposure, and shifting microbial colonisation toward aerotolerant bacteria such as *Rothia* species and *Cutibacterium acnes*.^14^^,^^45^ This ‘respiratory-driven’ microbial shift may reflect underlying physiological differences between ASD and neurotypical children and adults. Further research is required to determine whether mouth breathing in ASD is a consequence of anatomical, neurological, or behavioural factors or an interplay of these influences. Investigating its impact on oral microbiome composition, airway health, and neurodevelopmental outcomes could provide valuable insights into ASD-related microbial dysbiosis.

Another highly prevalent oral microbe noted in those with ASD is the *Bacillus* species. Kong et al^26^ noted a significantly higher prevalence of *Bacilli* species in both the oral and gut ecosystems of ASD individuals. This suggests that either shared environmental influences, such as diet, drive these microbial shifts, or the direct connectivity of the oral-gut microbial axis promotes the growth of such generic bacterial groups.

The latter research team^26^ also identified a strong correlation between gut *Firmicutes* and salivary *Chloroflexi* levels in ASD patients. Despite their disparate anatomical niches, this association suggests a systemic microbial interplay, where shifts in one microbial community are reflected in alterations in another distant site. These findings underscore a broader dysregulation of microbial networks in ASD, reinforcing the notion that the oral microbiome has far-reaching effects beyond the confines of the oral cavity. Similar correlations have been observed between oral and gut microbiomes in conditions such as irritable bowel syndrome and diabetes.^46^

### The oral microbiota in ASD: *Functional alterations and neurotransmitter dysregulation*

Changes in the microbiome have been associated with atypical communication patterns and repetitive behaviours in animal models. Microbiota from individuals with ASD induced altered social behaviour, atypical communication patterns and repetitive behaviours in the offspring of germ-free mice, with specific association between some of those behaviours and bacterial taxa.^47^ The present review highlights that the oral microbiota in children with ASD exhibits distinct functional alterations that may influence brain function and systemic physiology through multiple interconnected pathways.

Hicks et al^27^ examined the dysregulation of oral microbial RNA profiles, particularly in lysine degradation pathways, which has been linked to excessive glutamate production. Glutamate, a key excitatory neurotransmitter, plays an essential role in cognitive functions such as learning and memory. Under normal conditions, 75% to 95% of dietary glutamate is rapidly metabolised before reaching systemic circulation, ensuring tight homeostatic regulation and preventing the harmful accumulation of glutamate.^48^

Alterations in microbial lysine degradation pathways may also disrupt this balance, leading to increased permeability of the blood–brain barrier (BBB) and potentially allowing peripheral glutamate to enter the central nervous system.^49^ Elevated glutamate levels within the CNS have been reported in individuals with ASD, associated with synaptic dysfunction and neural hyperactivity.^50^ Such dysregulation may underlie core ASD symptoms, including cognitive impairments, sensory hypersensitivity, and repetitive behaviours. However, the reviewed relationship between microbial functional pathways and glutamate-mediated synaptic changes in ASD is currently correlational and not causative.

Additionally, the oral microbiota of children with ASD showed increased expression of bacterial transcripts associated with oxidative phosphorylation and methane metabolism. Elevated oxidative phosphorylation can lead to the overproduction of reactive oxygen species (ROS), contributing to systemic oxidative stress, neuronal damage, and mitochondrial dysfunction, ultimately affecting brain function and behavior.^51^^,^^52^

Moreover, methane metabolism in the oral cavity, though less common than in the gut, might indicate the presence of oral methanogenic bacteria. These bacteria can alter the local microbial ecology, promoting dysbiosis. Methane and other bacterial byproducts could have systemic effects, such as contributing to inflammation or influencing gut motility if swallowed,^53^ thereby indirectly affecting ASD-related behaviours.

The oral microbiome in children with ASD also exhibits distinct metabolomic activities associated with the degradation of key neurotransmitters, dopamine, and GABA. The latter may influence brain functionality and contribute to ASD pathophysiology. According to Manghi’s observation,^22^ microbial genes encoding dopamine and GABA degradation pathways are significantly enriched in the oral microbiomes of children with ASD. Dopamine is critical for reward processing, attention, and motor control,^54^ and its degradation by the oral microbiome could reduce its availability in the brain, potentially exacerbating ASD-related symptoms such as impaired motivation and attention deficits.

**Fig. 2:**
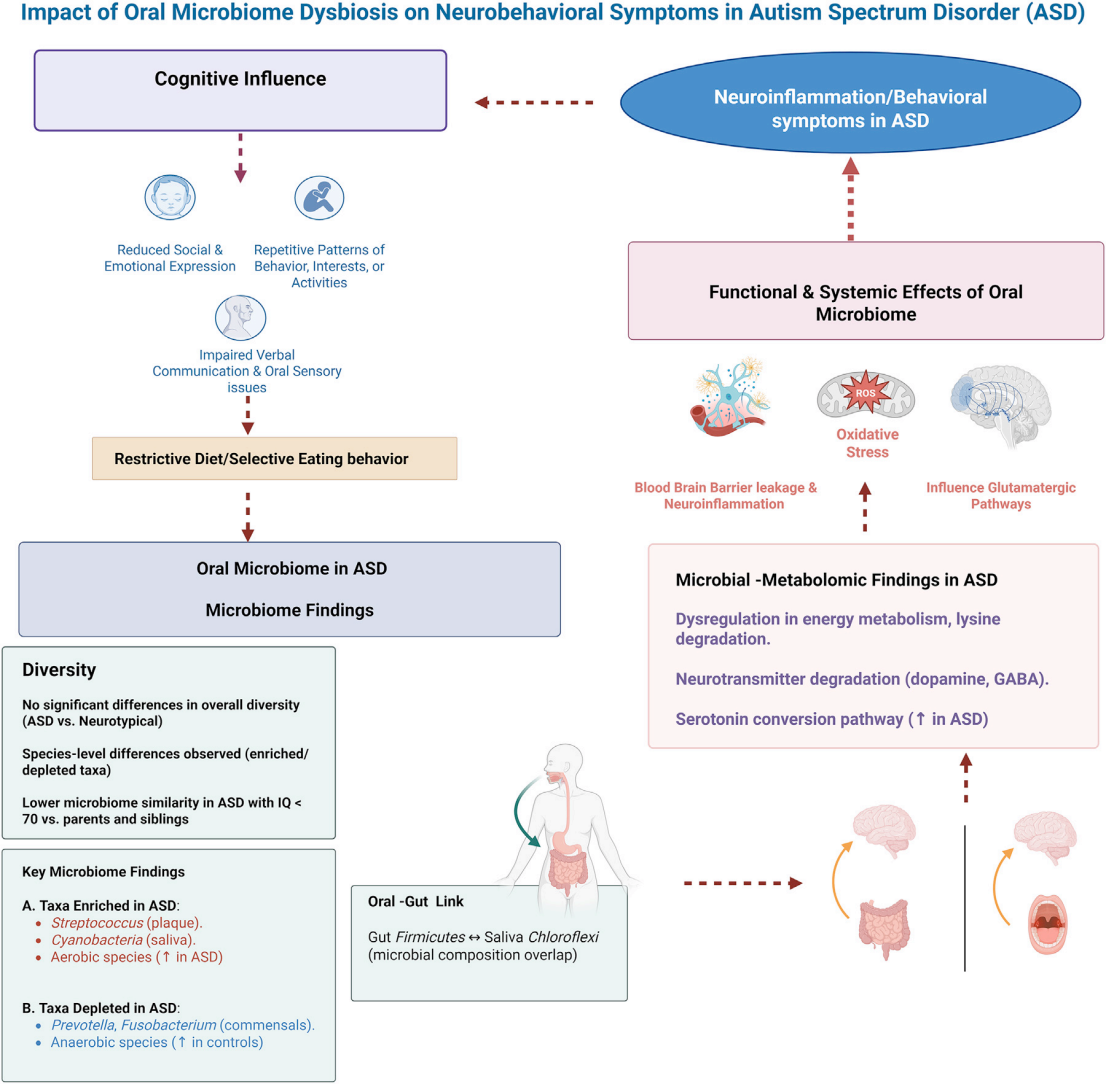
Impact of oral microbiome dysbiosis on neurobehavioral symptoms in autism spectrum disorder (ASD). This figure illustrates how cognitive and behavioural features of ASD, including restrictive eating and oral sensory issues, contribute to oral microbiome dysbiosis. Microbiome findings show species-level differences without significant overall diversity changes, and in ASD individuals with IQ < 70, reduced microbiome similarity to family members is observed. Oral dysbiosis may promote systemic and neurological effects through metabolic disruptions, oxidative stress, and neurotransmitter imbalances, contributing to neuroinflammation and reinforcing ASD symptoms in a feedback loop.

Similarly, GABA, the primary inhibitory neurotransmitter, plays a crucial role in neuronal excitability.^55^ Increased degradation of GABA may disrupt the excitatory-inhibitory balance in the brain, potentially contributing to sensory sensitivities, anxiety, and social communication challenges commonly observed in ASD. These findings underscore the potential role of the oral microbiome in modulating neurotransmitter levels, suggesting that microbial enzymatic activities could contribute to the neurochemical imbalances reported in ASD, as illustrated in Figure 2. This data lays the groundwork for further investigation into the oral microbiome’s impact on brain function in ASD and identifies potential therapeutic targets. However, causality cannot be inferred due to the observational nature of the included studies. Further mechanistic and longitudinal research is needed to explore how this microbial metabolome could influence cognitive development.

### Review limitations

While the reviewed studies provide valuable insights into the oral microbiome in ASD, there are significant inconsistencies in the reported findings. Some studies observed notable shifts in microbial composition, while others reported minimal differences between individuals with ASD and neurotypical controls. This heterogeneity underscores the need for a more nuanced interpretation of the current evidence and likely stems from multiple methodological and population-level factors.

First, considerable variability exists in sample collection procedures and analytical methodologies, which may account for the inconsistencies across studies (Table 2). Sampling sites varied, including saliva, tongue swabs, and dental plaque biofilm, each representing distinct microbial niches. In addition, differences in sequencing techniques likely influenced the depth and resolution of taxonomic identification. For example, while 2 studies employed whole genome sequencing, allowing species-level resolution, the others used 16S rRNA sequencing, which typically limits identification to the genus level. Furthermore, variations in DNA extraction methods, sequencing platforms, and bioinformatics pipelines were not consistently reported, adding another layer of experimental heterogeneity.

Second, population differences such as varying age ranges, geographic locations, and ASD phenotypes may have influenced microbial profiles. Lifestyle factors like diet, medication use, and oral hygiene practices were also inconsistently documented, yet these variables are known to significantly impact oral microbiome composition.

An additional confounding factor is the presence of gastrointestinal symptoms, reported in only some of the included studies. Given the interconnectedness of the oral and gut microbiota via the oral-gut axis, gastrointestinal disturbances could indirectly affect oral microbial composition, introducing further variability into the findings.

Another critical consideration is the selection of control groups. While most studies recruited neurotypical individuals from the general population, 2 studies used siblings or first-degree relatives of individuals with ASD as controls (Table 1). Prior research has shown that related individuals share more similar gut microbiota due to common environmental exposures, genetics, and dietary habits. Although this has not yet been explicitly confirmed for the oral microbiome in ASD, it is reasonable to hypothesise a similar pattern, making the inconsistent use of control groups a potential source of bias.

Moreover, the study by Manghi et al^22^ contributed the largest sample size, accounting for the majority of participants in this review. This disproportionate representation may have skewed the pooled findings, particularly those related to microbial diversity and species-level differences.

These methodological and population-level variations highlight the need for standardised protocols in future research, including uniform sample collection, consistent analytical methods, and better control of confounding factors. Larger, more diverse cohorts with detailed phenotyping of ASD symptoms are also essential to improve comparability across studies and to advance understanding of the role of the oral microbiome in ASD.

Importantly, there is a notable lack of robust correlation analyses between microbial profiles and core ASD symptoms, such as repetitive behaviours and communication impairments. This represents a critical gap in current literature. Future studies should integrate microbiome analysis with behavioural phenotyping to explore potential associations between specific microbial shifts and ASD-related clinical presentations.

Finally, only a limited number of studies have examined the oral microbiome metabolome in ASD. Current data are insufficient to determine whether functional microbial alterations contribute to ASD-related behaviours. Future research employing longitudinal designs and multi-omics approaches could elucidate the temporal and mechanistic relationships between microbiome shifts and ASD progression, providing deeper insights into the role of the oral microbiome in ASD pathophysiology.

## Conclusion

The inconsistent findings on oral microbial diversity in ASD highlight the critical need to move beyond cross-sectional studies. Future research must prioritise longitudinal investigations tracking microbial dynamics alongside ASD symptom progression from early childhood. This approach, coupled with robust multi-omics analyses and detailed behavioural phenotyping, is crucial for establishing causal relationships between specific microbial features and core ASD symptoms, such as repetitive behaviours and communication impairments, ultimately informing the development of targeted microbiome-based interventions.

## Author contributions

KSF and TP contributed substantially to the conception and design of the work and the acquisition, analysis, and interpretation of the data. IK, TM, AM contributed data analysis. LS and TP drafted and critically revised the work for important intellectual content. All authors critically revised the manuscript and gave final approval of the version to be published.

## Declaration of generative AI and AI-assisted technologies in the writing process

We used generative AI tools for grammar and spell check, which improved the language of this manuscript.

## Conflict of interest

The authors declare that they have no known competing financial interests or personal relationships that could have appeared to influence the work reported in this paper.
